# Effect of the Addition of Human Milk Fortifier to Breast Milk on the Early Recovery of Infants After Congenital Cardiac Surgery

**DOI:** 10.3389/fped.2021.661927

**Published:** 2021-04-27

**Authors:** Xian-Rong Yu, Wen-Peng Xie, Jian-Feng Liu, Li-Wen Wang, Hua Cao, Qiang Chen

**Affiliations:** ^1^Department of Cardiac Surgery, Fujian Maternity and Child Health Hospital, Affiliated Hospital of Fujian Medical University, Fuzhou, China; ^2^Fujian Key Laboratory of Women and Children's Critical Diseases Research, Fujian Maternity and Child Health Hospital, Fuzhou, China; ^3^Fujian Branch of Shanghai Children's Medical Center, Fuzhou, China; ^4^Fujian Children's Hospital, Fuzhou, China

**Keywords:** human milk fortifier, breastfeeding, infant, CHD, prognosis

## Abstract

**Objective:** This article studied the effect of breast milk supplemented with human milk fortifier (HMF) on the early recovery of infants after congenital cardiac surgery.

**Methods:** Infants undergoing congenital cardiac surgery were randomly divided into an intervention group (*n* = 27) and a control group (*n* = 27). Infants in the intervention group received HMF, and those in the control group were exclusively breastfed. The nutritional indicators at discharge, the postoperative recovery status, and nutritional-related complications were recorded.

**Results:** Compared with the control group at the time of discharge, the weight and albumin and prealbumin levels of the intervention group were significantly increased (*P* < 0.05). The length of hospital stay of the intervention group was significantly reduced compared with that of the control group (*P* < 0.05). Although the length of ICU stay for the intervention group was shorter than that of the control group, the difference was not significant (*P* > 0.05). No significant difference in the incidence of postoperative nutrition-related complications was noted between the two groups (*P* > 0.05).

**Conclusion:** Compared with breastfeeding alone, with HMF can improve postoperative weight gains, reduce the length of stay, and promote infants' early recovery after congenital cardiac surgery.

## Introduction

After congenital cardiac surgery, the nutritional status and nutritional intake of patients may be related to mortality and other clinical outcomes, and may lead to stunted growth, pulmonary infection, poor wound healing, the need for prolonged mechanical ventilation duration, and increased length of hospital stay (1, 2). Some patients with severe congenital heart diseases and moderate-severe pulmonary hypertension require surgical treatment during infancy as well as adequate calorie supplementation and strict water intake after surgery (3). Therefore, appropriate nutritional support is important for the early recovery of infants after congenital cardiac surgery. The standard enteral nutrition programs include breast milk and formula milk feeding programs. However, a single feeding plan may not balance the contradiction between postoperative energy needs and fluid restriction in infants. Based on the same breast milk intake, human milk fortifier (HMF) can increase energy density and alleviate this contradiction to a certain extent (4, 5). However, the effect of HMF on the early recovery of infants after congenital cardiac surgery remains unclear. We assumed that the careful introduction of HMF to breast milk might be beneficial to improve weight gain and potentially allow for earlier discharge of infants subjected to cardiac surgery. The purpose of this study was to evaluate the effect of breast milk supplemented with HMF on the early recovery of infants after congenital cardiac surgery.

## Methods

The ethics committee approved the present study (2020YJ181). The patients' parents were informed in detail about the content, procedure, and significance of the study, and provided informed consent.

The sample size of this study was determined as follows. According to the difference in the results of the two groups in a pilot study, we assumed that the difference between the two independent populations was 10% based on α = 0.05 and β = 0.1, suggesting 24 participants were required in each group. Assuming a loss to follow-up rate of 10%, the total sample size required was 54 cases (27 cases per group). This study was a prospective randomized controlled study conducted by a provincial teaching hospital on the southeast coast of China. The clinical data of 54 infants younger than 4 months who underwent cardiac surgery in our hospital were collected and analyzed from April 2020 to August 2020.

The inclusion criteria were as follows: 1. age <4 months; 2. suitability for breastfeeding; 3. good recovery of cardiac and circulatory functions after surgery; and 4. stable hemodynamics and satisfactory correction of intracardiac malformations; and 5. parents signed informed consent. The exclusion criteria were as follows: 1. premature infants; 2. severe pulmonary infection and other organ malformations before surgery; 3. emergency surgery; 4. severe congenital diseases of the gastrointestinal tract; 5. parents who declined to participate in the study; and 6. infants who were fed formula milk. The indications for early surgery were as follows: non-restricted ventricular septal defect or a life-threatening congenital cardiac malformation, ±moderate-severe pulmonary hypertension, repeated pulmonary infection, cardiac insufficiency, etc. No cardiac medications were used preoperatively, but vasoactive and cardiac medications were routinely administered postoperatively in all patients.

The patients were randomized into a control group and an intervention group. All infants included in the study were fed breast milk. Formula milk was not used in the study. Although breast milk came from different mothers, a team of professionals conducted a unified evaluation of the quality of breast milk and confirmed that the collected milk was clean and usable. The control group (*n* = 27) started feeding breast milk early after the operation, and HMF was gradually added to expressed breast milk given to the patients in the intervention group (*n* = 27) based on the practice of the early commencement of breast milk after the operation. The postoperative dietitian assessed whether the gastrointestinal function had recovered and provided a nutritional support program. Patients with tracheal intubation were first fed *via* a gastric tube and then gradually transitioned to normal feeding. When the amount of breast milk reached 80–100 ml/kg/d, HMF was added to the diets of intervention group patients to fortify the breast milk. HMF was produced by Nestlé, Germany. In the beginning, one-quarter of a gram of HMF was added to 25 ml of breast milk (25 ml breast milk: 0.25 g of HMF). After 3 days of good tolerance assessed by the dietitian, one-half of a gram of HMF was added to 25 ml of breast milk (25 ml breast milk: 0.5 g of HMF). Then, after another 3 days of acceptable tolerance, 1 gram of HMF was added to 25 ml of breast milk (25 ml breast milk: 1 g HMF). Finally, HMF was added at this fixed ratio and fed as needed until the patient was discharged.

### Data Collection

Preoperative data of the two groups of patients, such as age, sex, weight, different diagnoses of congenital heart diseases, pulmonary pressure, albumin, and prealbumin, were collected and recorded. Data on the intraoperative aorta clamp time, cardiopulmonary bypass time, whole operation time, length of ICU stay, breast milk intake during total enteral feeding, duration of different feeding methods, and length of postoperative hospital stay were also collected. Data on postoperative nutritional status indicators and nutrition-related complications at discharge, such as body weight, hemoglobin, albumin, and prealbumin, were recorded and analyzed.

### Statistical Analysis

SPSS 25.0 software was used for statistical analysis. Continuous data are presented as the mean ± standard deviation and range. The data were tested for a normal distribution and confirmed to be consistent with a normal distribution. Clinical parameters between the two groups were compared with the independent samples *t*-test. The χ^2^ or Fisher's test was used for categorical variables. A *p*-value < 0.05 was considered to be significant.

## Results

As shown in Table 1, 100 ml breast milk contained 68 kcal, and 100 ml breast milk after adding HMF contained 85 kcal. In addition, the content of protein, fat and trace elements were also increased in the intervention group. No statistically significant differences in terms of general preoperative data, preoperative nutritional status indicators, intraoperative aorta clamp time, cardiopulmonary bypass time, total operation time, breast milk intake or feeding methods were noted between the intervention and control groups (Table 2). Postoperative cardiac anatomic correction was satisfactory, and stable hemodynamics were noted in both groups. These results indicated that the data of the two groups were comparable. The quantity of breast milk intake during total enteral feeding is recorded in Table 3, and the results showed that the breast milk intake was comparable between the two groups.

**Table 1:** The main nutrients of the two milk formulas (per 100 ml).

**Nutrient content**	**Breast milk**	**Breast milk with HMF**
Calories (kcal)	68	85
Protein (g)	1.1	2.5
Fat (g)	3.8	3.8
Carbonic acid compound (g)	8	10.3
Calcium (mg)	34	100
iron (mg)	0	1.4
phosphorus (mg)	15	59
Vitamin A (IU)	189.8	441
Vitamin B (IU)	2	100

*HMF: human milk fortifier*.

**Table 2:** Comparison of general information of the two groups.

	**Intervention group**	**Control group**	***P-*value**
Age (month)	2.1 ± 1.2	2.4 ± 1.1	0.532
Weight (kg)	4.6 ± 0.8	4.5 ± 0.7	0.616
Pulmonary pressure	66.5 ± 8.2	63.3 ± 9.6	0.183
Diagnosis			
Ventricular septal defect	20	19	
Pulmonary stenosis	3	4	
Total anomalous pulmonary venous connection	1	1	0.850
Interrupted aortic arch	1	0	
Coarctation of aorta	2	3	
Albumin (g/L)	35.3 ± 4.1	36.2 ± 3.7	0.714
Prealbumin (mg/L)	151.5 ± 23.3	166.2 ± 26.2	0.525
Hemoglobin (g/L)	103.6 ± 12.2	106.5 ± 14.7	0.651
Operation time (h)	3.7 ± 1.3	3.8 ± 1.4	0.772
Cardiopulmonary bypass time (h)	1.8 ± 0.8	1.9 ± 1.0	0.837
Aortic clamp time (h)	0.9 ± 0.4	0.8 ± 0.5	0.846

**Table 3:** Comparison of the postoperative situation of the two groups.

	**Intervention group**	**Control group**	***P*-value**
Breast milk intake (ml/kg.d)	152.2 ± 10.8	153.6 ± 8.8	0.629
Feeding method			
Duration of gavage feeding (days)	3.1 ± 1.0	3.3 ± 1.2	0.325
Duration of bottle-feeding (days)	2.6 ± 1.3	2.9 ± 1.5	0.279
Duration of breastfeeding (days)	7.5 ± 2.1	8.0 ± 2.3	0.398
The length of ICU stay (d)	5.0 ± 2.1	5.8 ± 2.9	0.346
The length of postoperative hospital stay (d)	13.3 ± 3.2	15.2 ± 4.1	0.043
Weight at discharge (kg)	5.3 ± 1.0	4.7 ± 0.8	0.041
Albumin at discharge (g/L)	48.2 ± 4.2	41.1 ± 5.4	0.033
Prealbumin at discharge (mg/L)	221.3 ± 25.1	187.3 ± 29.5	0.021
Hemoglobin at discharge (g/L)	133.1 ± 13.6	129.6 ± 15.2	0.429

Comparing postoperative nutritional indicators of the two groups at discharge, the results showed that the weight and albumin and prealbumin levels of the intervention group were significantly higher than those of the control group (*P* < 0.05). Compared with the control group, the length of hospital stay of the intervention group was significantly shorter (*p* < 0.05). Although the length of ICU stay was shorter for the intervention group compared with the control group, the difference was not statistically significant (*P* > 0.05). Regarding postoperative nutrition-related complications, there were no significant differences in the incidence of postoperative pneumonia, liver insufficiency, gastrointestinal bleeding, feeding intolerance, necrotizing enterocolitis, or jaundice between the two groups (Table 4).

**Table 4:** Comparison of complications between the two groups.

	**Intervention group**	**Control group**	***P*-value**
Postoperative pneumonia	3	3	1.0
Necrotizing enterocolitis	0	0	-
Liver insufficiency	2	3	0.639
Gastrointestinal bleeding	0	0	-
Feeding intolerance	3	2	0.639
Jaundice	1	0	-

## Discussion

After cardiac surgery and cardiopulmonary bypass, the immediate postoperative period is characterized by an intense stress response with activation of the immune-neuroendocrine axis and inflammatory cascade (6, 7). Then, gastrointestinal tract complications, such as gastrointestinal dysfunction, gastrointestinal bleeding, bloating, diarrhea, indigestion, and necrotizing enterocolitis, may occur and impede nutrient absorption. At present, perioperative management to meet the nutritional needs of infants after congenital cardiac surgery represents a serious challenge (8, 9). In addition, infants may have persistent cardiac dysfunction and fluid retention after cardiac surgery, making it necessary to strictly limit the amount of fluid intake to reduce the workload of the heart (10). Therefore, attempts to increase the energy density of breast milk as much as possible in a setting of fragile gastrointestinal function so as to be tolerated after cardiac surgery was the focus of our current perioperative management.

Breast milk is the best option to provide nutrition for infants. Considerable evidence has demonstrated that breast milk has a beneficial effect on the nutritional status, immunity level, digestive function, growth factor content, and neurodevelopment of preterm infants. (11–13). Infants require higher nutritional intake after cardiac surgery to supplement energy and promote rapid tissue proliferation. However, the relatively low energy density in breast milk, especially protein, may not meet the perioperative infants' needs (14, 15). The protein content of breast milk is reduced a week after birth and may be too low to meet infants' protein needs during the perioperative recovery phase (16, 17). In particular, after congenital cardiac surgery, infants are further affected by the surgical trauma and cardiopulmonary bypass (8, 9). Their energy needs are greater than those infants after general surgery. Therefore, breastfeeding alone may not meet the energy needs of such infants. HMF contains various proteins, carbohydrates, minerals, and vitamins, which can significantly increase breast milk energy density, strengthen and optimize protein intake, and promote physical growth (18, 19). Studies have shown that HMF may increase the energy intake and nutritional status of preterm infants (18, 19). However, few studies have focused on the effect of HMF on the early recovery of infants after congenital cardiac surgery.

In this study, HMF was applied to promote the early recovery of infants after congenital cardiac surgery. Although there was no difference in the total breast milk intake of the two groups, the HMF provided the infants with more energy and other nutrient element intake. The study showed that the weight as well as albumin and prealbumin levels of the intervention group at discharge were significantly greater than those of the control group, which confirmed that HMF could improve the postoperative weight gains and nutritional status after surgery. Simultaneously, the length of hospital stay of the intervention group was reduced compared with that of the control group, indicating that HMF could speed up the early recovery of these infants. After congenital cardiac surgery, infants' gastrointestinal function was negatively affected by many factors, including gastrointestinal ischemia, reperfusion injury after cardiopulmonary bypass, postoperative mechanical ventilation, and vasoactive drugs (20). In addition, HMF had a higher osmotic pressure, which would increase the risk of gastrointestinal complications, such as necrotizing enterocolitis and feeding intolerance (21–23). Therefore, many researchers are worried that HMF might increase postoperative gastrointestinal complications. In our study, we gradually added the full amount of HMF based on the patients' gastrointestinal function recovery after cardiac surgery. No significant differences in the incidence of nutrition-related complications were noted between the two groups. These results also indicate that HMF would not increase postoperative gastrointestinal complications.

This study had some limitations. We studied the effect of breast milk supplemented with HMF on the early recovery stage of infants after cardiac surgery and did not use formula milk as the control group, so our conclusion could not be generalized to patients who used formula milk. Further studies need to be performed to confirm whether formula milk with HMF has the same effect in this group of patients. This study was a single-center study with a small sample size, and there may be deviations in the selection of cases. The research indicators adopted in this study were limited, which may affect the judgment of the research results. In addition, this paper was not a double-blind study, which might also affect the accuracy of the results. We hope to complete a randomized, controlled, prospective, double-blind study with a larger sample size in the future to further reinforce our conclusions.

## Conclusion

For infants after congenital cardiac surgery, breast milk supplemented with HMF can increase the energy intake, improve postoperative weight gains, reduce the length of stay, and promote early postoperative rehabilitation.

## Data Availability Statement

The original contributions generated for the study are included in the article/supplementary materials, further inquiries can be directed to the corresponding author/s.

## Ethics Statement

The studies involving human participants were reviewed and approved by Fujian Maternity and Child Health Hospital, Affiliated Hospital of Fujian Medical University. Written informed consent to participate in this study was provided by the participants' legal guardian/next of kin.

## Author Contributions

X-RY and QC designed the study, performed the statistical analysis, participated in the operation, and drafted the manuscript. J-FL, W-PX, L-WW, and HC collected the clinical data. All authors read and approved the final manuscript.

## Conflict of Interest

The authors declare that the research was conducted in the absence of any commercial or financial relationships that could be construed as a potential conflict of interest.
